# Influence of Speed and Rainfall on Large-Scale Wheat Lodging from 2007 to 2014 in China

**DOI:** 10.1371/journal.pone.0157677

**Published:** 2016-07-01

**Authors:** Liyuan Niu, Suwei Feng, Weihua Ding, Gan Li

**Affiliations:** 1 College of Life Science and Technology, Henan Institute of Science and Technology, Xinxiang, Henan, China; 2 Wheat Breeding Institute, Henan Institute of Science and Technology, Xinxiang, Henan, China; Institute of Genetics and Developmental Biology, CHINA

## Abstract

Strong wind and heavy rain remain the two most important causes of large acreage wheat (*Triticum aestivum* L.) lodging in China. For research the influence of wind speed and rainfall—separately as well as together—on the extent and degree of lodging, five levels of the severity of lodging were defined based on a combination of the lodging area and the degree of tilting. Detailed meteorological information was studied on 52 instances of large-scale lodging that occurred from 2007 to 2014. The results showed that strong wind’s lodging accounted for 8% of the instances studied, continuous rainfall’s lodging accounted for 19% and strong winds-heavy rainfall’s accounted for 73%. The minimum instantaneous wind speed that could cause large-scale lodging was closely related to rainfall. Without rainfall, the wind speed that resulted in lodging ranging in severity from slight to severe (Level 2 to Level 5) was 14.9 m/s, 19.3 m/s, 21.5 m/s, and 26.5 m/s, respectively; when accompanied by rainfall, the wind speed that resulted in lodging of the same severity decreased linearly with the increase of rainfall. These results will be particularly useful in preventing and alleviating wheat lodging as well screening wheat varieties with good lodging resistance.

## Introduction

Lodging, or the permanent tilting or bending of stems from the vertical, is a major limiting factor to grain production worldwide [1]. China is the world’s largest producer of winter wheat (*Triticum aestivum* L.), and lodging, in severe cases, can lower the wheat production by as much as 80% [2] besides being a potential health risk in the form of fungal infection of grains and the subsequent development of mycotoxins [3–5]. Lastly, lodging also makes mechanical harvesting difficult [2, 5]. In China, food production lost due to lodging was estimated at more than 2 million tones annually [2]. Therefore, a detailed study of the influence of the wheat plant itself and of meteorological factors on lodging and its mechanism is essential to devise practical strategies to reduce crop losses due to lodging and to further our understanding of the theoretical aspects of lodging.

Recent years have seen many papers on lodging in wheat, which is generally related to a variety of factors, such as the environment [1], management practices, the period of growth and development [1,6–7], and varietal characteristics [6,8–9]. Among these, wind and rain are the two most important factors [1]. Because lodging occurs at random and cannot be simulated, besides being difficult to quantify and to observe systematically, little research has been conducted so far on the influence of wind speed and rainfall—separately as well as together—on the extent and severity of lodging. However, in recent years, the extensive telecommunication network and news coverage make it easier to collect information on instances of large-scale lodging. The present paper draws on such network news and meteorological data from 2007 to 2014 to study such relevant factors as wind speed, rainfall, and growth stages of wheat and the impact of these factors on the extent and severity of lodging to guide further theoretical research on lodging and for screening wheat varieties for resistance to lodging.

## Research Methods

### Lodging in wheat and historical weather data

Information about instances of large-scale lodging in wheat from 2007 to 2014 was collected from the public reports on the Internet, and indicated the specific source in the references. This information, in the form of text and images (including video clips), was refined by adding the exact location, date, and the severity of lodging by means of further searches, electronic maps, databases, and so on. The final list comprised 52 instances of lodging for each of which sufficient data were available, including the stage of the crop, wind speeds, and the intensity of rainfall. In particular, we consulted two large data sets, namely the ‘China terrestrial climate data daily value data sets’ [10] and the ‘China daily precipitation grid real-time analysis system (Version 1.0) data set’ [11], obtained from the China meteorological data sharing network (http://cdc.cma.gov.cn). All the data on rainfall and wind speed from the news reports and weather forecasts were checked and verified against these data sets. Whenever necessary, the data on wind speed and rainfall were either obtained from a China national benchmark meteorological observatory station nearest to the site of lodging or were interpolated from such data. To make it easier to compare the data, the wind speed used was the average maximum wind speed (instantaneous maximum wind speed) 10 m above the ground for 3 seconds recorded from 8 p.m. to 8 p.m. of the next day. The Beaufort scale by literature or the weather forecast took the median of its corresponding wind speed scale (m/s) as the maximum average wind speed for 10 minutes, then converted to the instantaneous maximum wind speed multiplied by 1.59; Rainfall was the daily rainfall recorded from 8 p.m. to 8 p.m. of the next day.

### Growth stages of wheat

The entire growth period of wheat was divided into ten stages based on the Zadoks’ cereal development scale [12]. Because lodging occurs mainly from the anthesis stage to the maturity stage, the study was mainly confined to three of the ten growth stages (GS), namely anthesis, milk development, and maturity. The anthesis stage extends from the beginning of flowering to full flowering (GS 60–69); the grain filling stage extends from the beginning starch deposition in the grains to endosperm in the condensed form (GS 70–79); and the maturity stage extends from the point at which the grain begins to harden to the point at which it is too hard to be split with a thumbnail (GS 80–91).

### Degree of severity of lodging

To facilitate computerized analysis of data, the severity of lodging was divided into five levels or degrees based essentially on China’s national agricultural industry standard NY/T1301-2007 ‘Technical procedures for wheat variety regional trials’[13] That standard was supplemented by including the extent (area) of lodging as a percentage of the area under wheat. The levels were as follows.

Level 1: no lodging
Level 2: slight lodging. Lodging area less than 20% of the total area under wheat at the given site; stems tilted at an angle less than 30°
Level 3: medium lodging. Lodging in small, scattered patches; lodging area 20%–40% of the total area; tilt angle 30°–45°
Level 4: heavy lodging. Lodging in large but scattered patches, lodging area 40%–80% of the total area; tilt angle 45°–60°
Level 5: severe lodging. Lodging in large, contiguous areas; lodging area more than 80% of the total area; tilt angle more than 60°

### Statistical analysis

All statistical analysis was carried out using SPSS ver. 13.0 (SPSS Inc., Chicago, IL, USA).

## Results

Details of each of the 52 instances of lodging chosen for analysis are given in Table 1. The data include the location, date, and time; rainfall and maximum wind speed; the growth stage of wheat at which the crop was affected; the level of lodging (on a scale of 1, no lodging to 5, severe lodging); and one or more supporting references.

**Table 1:** Large-scale lodging in winter wheat in China: date, location, rainfall, wind speed, growth stage of crop, severity and area.

No.	Date	Location and province	Rainfall (mm)^a^	Wind speed (m/s)^b^	Growth stage^d^	Lodging level, area and data sources
1	05/22/2007	Qufu county, Shandong	66.4^c^	10.8–13.3	GFS	Level 4, lodging mostly along the wind direction, 80,667 ha [14]
2	05/28/2007	Tanching county, Shandong	<1.0	24.4	GFS	Level 5, 533 ha [15]
3	06/04/2008	Yinan county, Shandong	58.5^c^	17.2	MS	Level 4, large area, irregular lodging [16]
4	05/17/2008	Weishi county, Henan	52.4	26.5	GFS	Level 5, lodging along the wind direction, 12,000 ha [17]
5	06/12/2009	Huocheng county, Xinjiang Uygur Autonomous Region	21.3	17.2	GFS	Level 4, lodging along the wind direction, 1,300 ha [18]
6	06/06/2009	Luyi county, Henan	50.0	22.6	MS	Level 5, complete lodging along wind direction, 1,300 ha [19]
7	06/03/2009	Xiayi and Yongcheng counties, Henan	23.2	29.1	MS	Level 5, complete lodging roughly in one direction, 200,000 ha [20]
8	06/08/2010	Suzhou city, Anhui	72.5	16.2	MS	Level 4, complete lodging in one direction,121,000 ha [21]
9	06/22/2010	Kuitun city, Xinjiang Uygur Autonomous Region	30.0	30.1	GFS	Level 5, complete lodging in one direction, 1000 ha [22]
10	06/18/2010	Heze City, Shandong	20.0	17.7	MS	Level 5, large-scale lodging in one direction [23]
11	05/30/2010	lingbi county and Si county, Anhui	34.4	26.5	MS	Level 5, large-scale lodging only in one direction, 40,000 ha [24–25]
12	06/06/2011	Jize county, Hebei	23.9	19.9	MS	Level 4, large-scale lodging in one direction [26]
13	06/06/2011	Neiqiu county, Luquan city in Hebei	23.2	30.6	MS	Level 5, incomplete lodging in one direction, 2,200 ha [27]
14	04/12/2012	Jingzhou, Jiangling and Gong'an county, Hubei	22.0–46.0	15.4	AS	Level 3, lodging area 30,000 ha [28]
15	05/16/2012	Lingbi county, Anhui	0.9	26.5	GFS	Level 5, large-scale irregular lodging [29]
16	06/10/2012	Jiyang county, Shandong	54	22.6	MS	Level 5, lodging area 5,300 ha [30]
17	05/07/2012	Langxi county, Anhui	82.2	9.1	GFS	Level 2, lodging in one direction but in strips [31]
18	07/02/2011	Altay city, Xinjiang Uygur Autonomous Region	28.1	14.9	GFS	Level 4, large-scale lodging along the wind direction, 50 ha [32]
19	06/02/2012	Shouguang city, Shandong	30.0	17.2	MS	Level 5, complete lodging along the wind direction[33–34]
20	06/26/2012	Wulatezhongqi, Inner Mongolia Autonomous Region	80.2–171.0	11.9–17.2	GFS	Level 5, large-scale lodging over a contiguous area, 8,500 ha[35]
21	05/25/2012	Lijin county,Shandong	4.3	14.1	GFS	Level 2, stems tilted in one direction to less than 30° [36]
22	06/07/2012	Linyi county, Shandong	34.4	19.0	MS	Level 5, complete lodging along the wind direction, 2,000 ha [37]
23	06/06/2012	Yutian county, Hebei	15.3	14.4	GFS	Level 2, small area, irregular lodging, 2,000 ha [38]
24	05/25/2013	Weihai city, Shandong	48.7	16.3	GFS	Level 3, patchy lodging, 6,600 ha [39]
25	05/24/2013	Boxing county, Shandong	25–50	10.6	GFS	Level 2, patchy lodging in a radiating pattern [40–41]
26	05/27/2013	Dong 'e county, Shandong	20.0	26.5	GFS	Level 5, large-scale lodging in one direction [42–43]
27	05/25/2013	Liaocheng city, Shandong	114.3	11.8	GFS	Level 4, large-scale lodging in one direction, 23,000 ha [44]
28	05/26/2013	Huantai county, Shandong	30.0	11.1	GFS	Level 3, lodging partly along the sowing direction [45]
29	05/25/2013	Chiping county, Shandong	46.7	19.0	GFS	Level 4, large-scale lodging in one direction(Wu, 2013)[46]
30	05/25/2013	Qufu city, Shandong	109.0	10.9	GFS	Level 5, large-scale lodging along the wind direction [47–48]
31	07/15/2013	Nenjiang county, Heilongjiang	54.7	18.9	AS	Level 5, large-scale and complete lodging [49]
32	05/25/2013	Shanghe county, Shandong	44.2^c^	14.9	GFS	Level 2, point, patchy lodging [50]
33	05/25/2013	Yinan county, Shandong	101.3^c^	15.3	GFS	Level 4, large-scale lodging in one direction [51]
34	05/27/2013	Zaozhuang city, Shandong	109.4	12.3	GFS	Level 5, large-scale continuous lodging [52–53]
35	05/22/2013	Yuncheng city, Shanxi	40.7	23.3	GFS	Level 5, large-scale lodging in one direction, 1,100 ha [54]
36	05/26/2013	Dezhou city and NingJin county, Shandong	25.2–62.9	25.2	GFS	Level 4, large-scale lodging [55–56]
37	05/25-26/2013	Ningyang county, Shandong	104.0^c^	14.9	GFS	Level 4, large-scale lodging in one direction [57]
38	05/25-26/2013	Yancheng city, Jiangsu	30.0	19.7	GFS	Level 4, large-scale lodging in one direction [58]
39	05/26/2013	Dafeng city, Jiangsu	40.0	16.6	GFS	Level 4, large-scale, irregular lodging [59]
40	05/25/2013	Pizhou county, Suining county, Jiangsu	100.0^c^	15.5	GFS	Level 4, patchy lodging, 16,000 ha [60–61]
41	06/02/2013	Daming county, Hebei	23.3	19.0	MS	Level 5, large-scale lodging in one direction, 4,000 ha [62–63]
42	06/25/2013	Botou city, Hebei	40.0^c^	14.9	MS	Level 4, large-scale, irregular lodging [64]
43	05/25/2013	Jingxi county, Hebei	32.0	17.2	GFS	Level 3, patchy, incomplete lodging in one direction [65]
44	05/26/2013	Wuqiao county, Hebei	32.0	17.2	GFS	Level 4, lodging along the planting direction, 1,000 ha [66]
45	06/07/2013	Zhengding county, Hebei	76.5	17.2	MS	Level 4, lodging area 6,600 ha [67–68]
46	06/09/2013	Longyao county, Hebei	35.3	19.0	MS	Level 4, 80% lodging in one direction [69–70]
47	05/24/2013	Wuyang city, Henan	77.0	13.8	GFS	Level 5, large-scale lodging in one direction [71–72]
48	05/25/2013	Xinxiang city, Henan	66.5^c^	12.6	GFS	Level 3, patchy, incomplete lodging in one direction [73]
49	04/05/2013	Jingzhou city, Hubei	7.8	18.3	AS	Level 3, patchy lodging, 17,000 ha [74]
50	05/22/2013	Yongji city and Jjishan county, Shanxi	21.2	21.3	GFS	Level 4, lodging in strips in one direction, 11,550 ha [75–76]
51	05/26/2013	Heze city, Shandong	86.4	16.4	GFS	Level 4, large-scale lodging in one direction, 6,600 ha [77]
52	05/26/2013	Weifang city, Shandong	11.7	15.3	GFS	Level 3, large-scale lodging in one direction [78]

^***a***^ Rainfall (mm): the daily rainfall between 8 p.m. to 8 p.m. of the next day.

^***b***^ Wind speed (m/s): the average maximum wind speed on the same day at 10 m from the ground and prevailing for at least 3 seconds.

^***c***^ Rainfall type: the continuous rainfall.

^***d***^ Growth stage: AS = Anthesis stage; MS = Mature stage; GFS = Grain filling stage.

### Lodging time and region distribution

The data in Table 1 show a more or less continual increase in the frequency of lodging: 2007 and 2008 saw two instances of lodging each; 2009 and 2011 saw three instances each; 2010, four; 2012, nine; and 2013, twenty-nine. The instances were distributed in the nine provinces or autonomous regions of Henan, Hebei, Shandong, Shanxi, Anhui, Jiangsu, Hubei, Xinjiang and Inner Mongolia.

### Growth stages and lodging

Among the three stages during which lodging was observed, the instances were distributed as follows: anthesis, 2 instances; milk development, 35 instances; and maturity, 15 instances.

### Meteorological factors and lodging types

The instances of lodging fell into three categories by cause, namely strong wind, continuous rain, and strong wind coupled with heavy rain.

#### Strong wind and lodging

**Fig 1:**
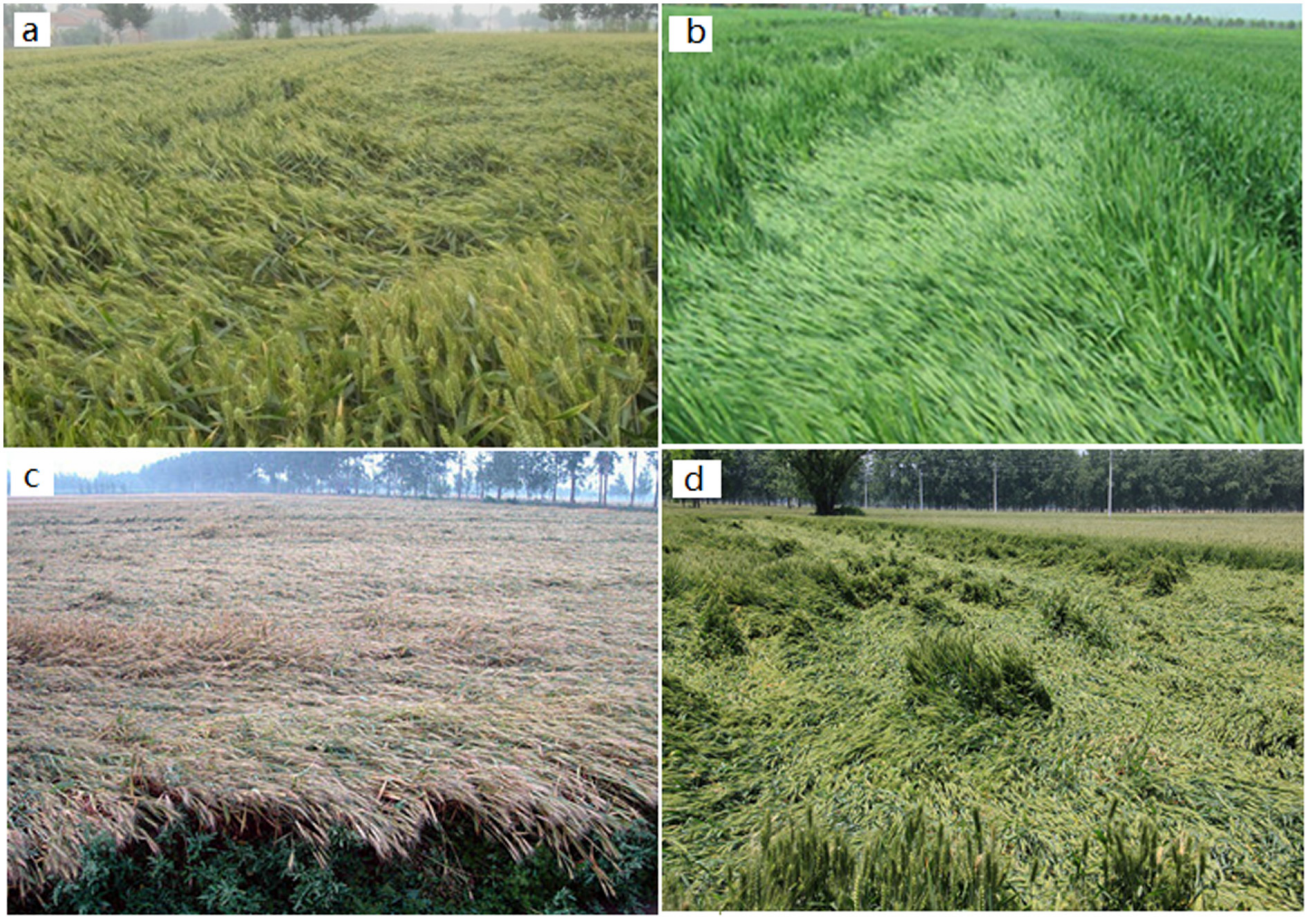
Lodging due to strong wind. a. Lijin county, Shandong (05/25/2012). b. Jingzhou city, Hubei (04/05/2013). c. Tancheng county, Shandong (05/28/2007). d. Lingbi county, Anhui (05/16/2012).

Strong wind was taken as the cause of lodging in those instances where strong wind was the sole cause, that is when it was not accompanied by any rainfall or accompanied by only slight rainfall (< 10.0 mm). Among the 52 lodging events studied, only four (Fig 1a–1d) fell in this category: in 2007 in Tancheng county, Shandong; in 2012 in Lijin county, Shandong and in Lingbi county, Anhui; and in 2013 in Jingzhou city, Hubei. Strong winds could lead to lodging at all levels of severity over large areas.Wind at 14.1 m/s resulted in Level 2 lodging in Lijin; that at 18.3 m/s resulted in Level 3 lodging over 17 000 ha in Jingzhou; and that at 24.4 m/s resulted in Level 5 lodging over more than 600 ha in Tangcheng. One obvious characteristic of such lodging was that stems of all the plants had tilted in the same direction, namely along the direction of wind.

#### Continuous rainfall and lodging

**Fig 2:**
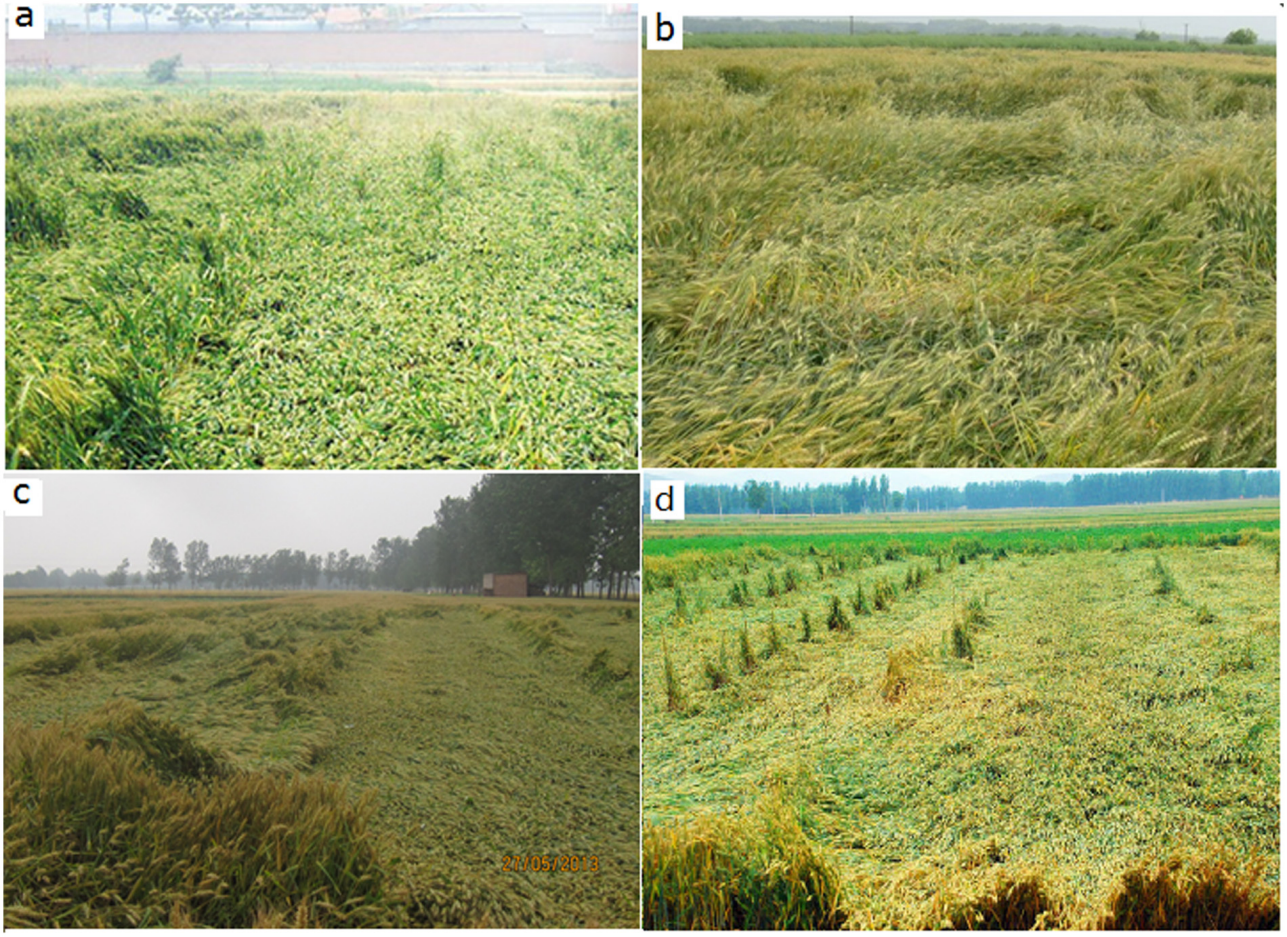
Lodging due to continuous rainfall. a. Jining city, Shandong (05/22/2007). b. Langxi county, Anhui (05/08/2012). c. Xinxiang city, Henan (05/26/2013). d. Zaozhuang city, Shandong (05/26/2013).

Continuous rainfall in the present context refers to rains lasting from several hours to several days at a time and a high rainfall intensity but not accompanied by strong winds. Of the 52 lodging events, 10 fell into this category including, for example, the lodgings that occurred in Jining city, Shandong, in 2007 (Fig 2a); Langxi county, Anhui, in 2012 (Fig 2b); Xinxiang city, Henan, in 2013 (Fig 2c); and Zaozhuang city, Shandong, in 2013 (Fig 2d). The most important features of lodging due to rainfall were that (1) the lodging was messy, the plants lodging randomly in different directions and (2) more often, the plants had lodged from the root plate. The severity of lodging varied from slight to severe.

#### Strong winds accompanying heavy rainfall and lodging

The most common cause of lodging—observed in 73% of the lodging events that constituted the present study—was a combination of strong winds and heavy rainfall. Depending on the wind speed and the intensity of rainfall, the severity of lodging ranged from Level 3 to Level 5.

**Fig 3:**
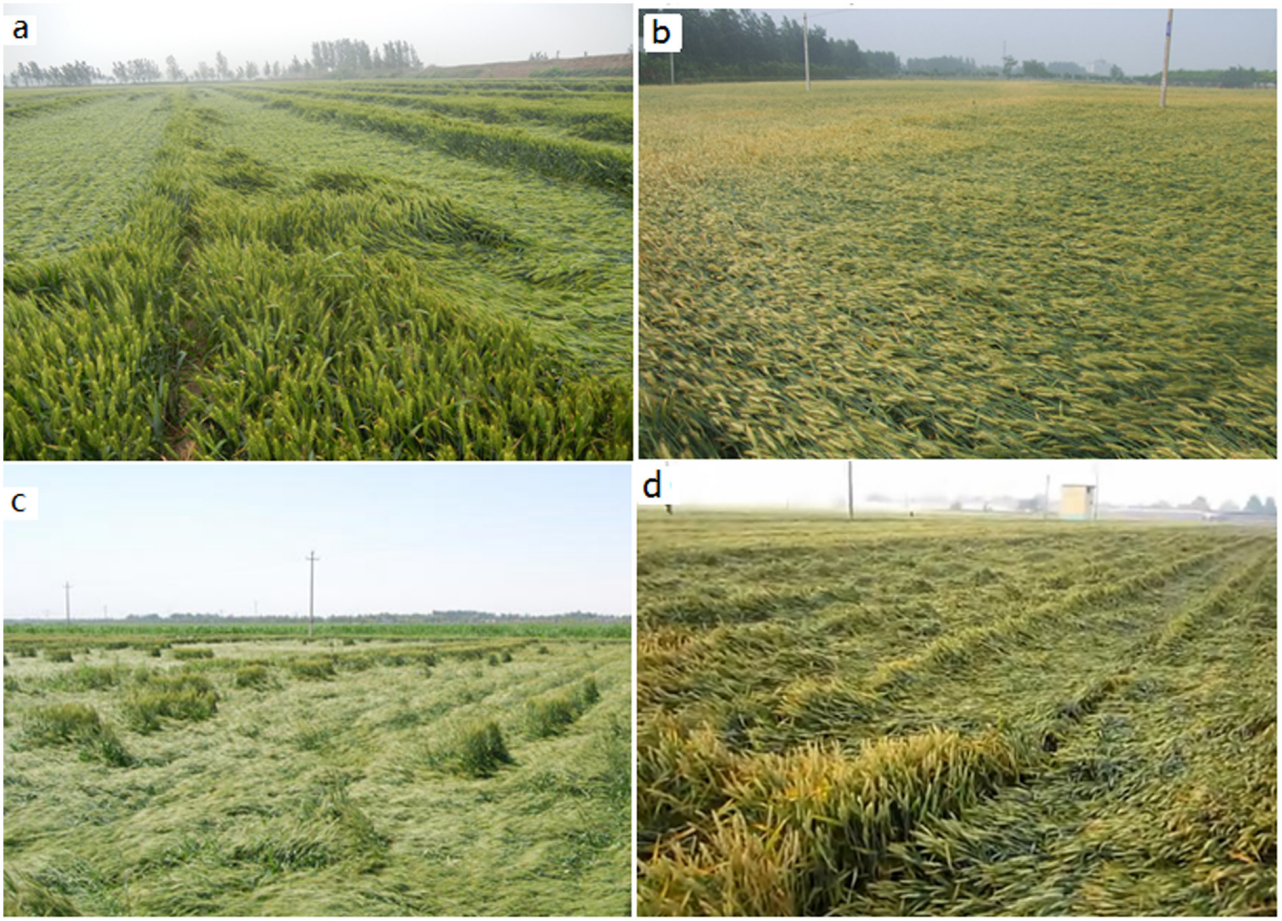
Lodging due to strong winds accompanied by heavy rainfall. a. Chiping county, Shandong (05/27/2013). b. Weishi county, Henan (05/17/2008). c. Wulatezhongqi, Inner Mongolia Autonomous Region (06/28/2012). d. Shouguang city, Shandong (06/02/2012).

The most important feature of lodging due to a combination of strong winds and heavy rain was that the lodging occurred in the downwind direction. Depending on whether the wind direction and the planting direction were at right angles to each other or parallel, the lodging was divided into two typical types. When the two directions were at right angles to each other, the lodging was extensive and along the direction of the wind, which is illustrated by the lodging in Chiping county, Shandong, in 2013 (Fig 3a) and that in Weishi county, Henan, in 2008 (Fig 3b). When the wind direction was parallel to the planting direction, the lodging occurred in surges, or waves, which is illustrated by the lodging in Shouguang city, Shandong (Fig 3c) and in Wulatezhongqi in Inner Mongolia. Both in 2012 (Fig 3d).

## Discussion

### Analysis of meteorological factors

#### Wind speed, rainfall, and severity of lodging

To study the effect of instantaneous maximum wind speed, singly or together with rainfall, on the degree of severity of lodging, the data were analyzed using the method of least significant difference (Table 2).

**Table 2:** Wind speed, rainfall, and severity of lodging in wheat.

Severity of lodging (Level)	No. of instances(n = 52)	Instantaneous maximum wind speed (m/s)			Daily rainfall (mm)		
		Minimum	Maximum	Mean±SD	Minimum	Maximum	Mean±SD
2	4	9.1	14.4	12.1 ± 2.3a*	4.3	80.0	34.3 ± 29.0a
3	8	11.1	18.3	14.8 ± 2.7ab	7.8	59.0	33.4 ± 16.4a
4	20	11.8	21.3	16.8 ± 2.3b	21.3	114.3	57.6 ± 29.0a
5	20	12.3	30.6	22.0 ± 5.2c	0.0	125.6	45.6 ± 34.2a

*LSD test; *p* = 0.05

As can be seen in Table 2, the severity of lodging and the instantaneous maximum wind speed and/or daily rainfall was positively correlated. Except for level 3 lodging, the instantaneous maximum wind speed in other different degrees of lodging had significant differences (*P*<0.05), but no significant differences in daily rainfall (*P>*0.05).

**Fig 4:**
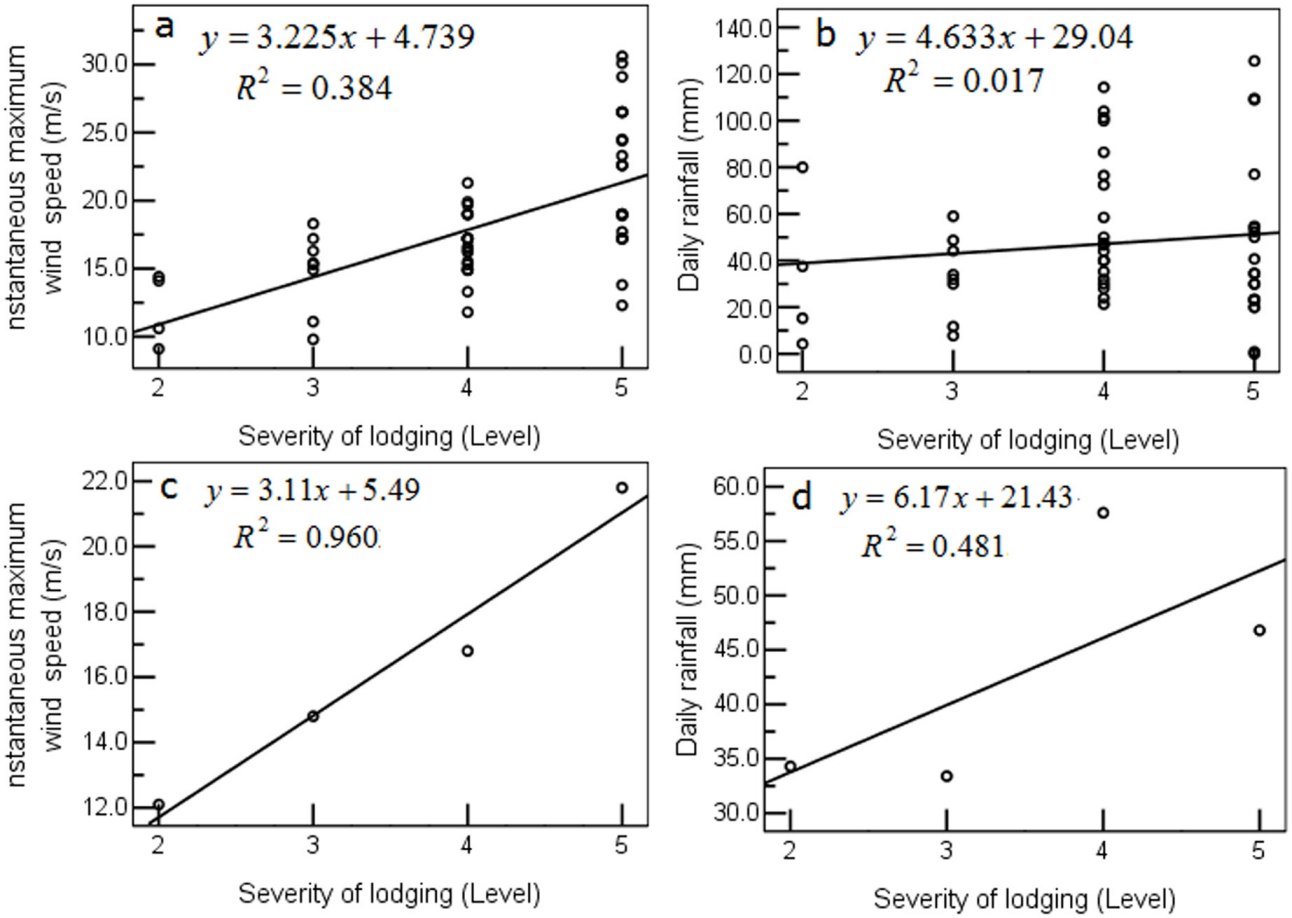
Effect of instantaneous maximum wind speed and rainfall on the severity of lodging in wheat. a. Effect of sample instantaneous maximum wind speed. b. Effect of sample rainfall. c. Effect of sample average instantaneous maximum wind speed. d. Effect of sample average rainfall.

The most common cause of lodging was the combined action of wind and rain. The two varied a great deal and yet led to the same degree of lodging (Fig 4a and 4b), which makes it difficult to isolate the contribution of each. Therefore, we plotted the mean values of wind speed and rainfall against the level of severity of lodging and developed appropriate linear equations (Fig 4c and 4d). The statistics showed that sample instantaneous maximum wind speed and sample average instantaneous maximum wind speed and the severity of lodging were significantly positively correlated, correlation coefficient r were 0.624 (*P*<0.01, *R*^*2*^ = 0.389) (Fig 4a) and 0.980 (*P*<0.05, *R*^*2*^ = 0.960) (Fig 4d), respectively; and sample daily rainfall and sample average daily rainfall and the severity of lodging were not correlated, correlation coefficient were 0.132 (*P* = 0.349>0.05, *R*^*2*^ = 0.017) (Fig 4b) and 0.694 (*P* = 0.306>0.05, *R*^*2*^ = 0.481) (Fig 4d). Wind speed was the dominant factor in lodging whereas rainfall was the auxiliary factor. The combined effect, which was significant (although the effect of wind speed was greater), of instantaneous maximum wind speed and daily rainfall on the degree of lodging can be expressed by the following regression equation:

$$y=0.146x_{1}+0.013x_{2}+0.146$$

Where *x*_1_ is the wind speed, *x*_2_ is the daily rainfall, *y* is the degree or severity of lodging, F = 32.509, and *P* = 0.000 < 0.01.

#### Rainfall and lodging

At present, it is generally believed that lodging in wheat is caused more by the bending moment induced by wind at the stem base or on the root plate than by the failure moment of the base stem or the root plate [1]. Although rainfall was not the main cause of large-scale lodging, rainfall significantly affected the severity of lodging by two ways: (1) rainfall can increase the weight of wheat plants to reduce the failure moment of the stem base and indirectly reduce the critical wind speed of wheat lodging; (2) rainfall can reduce the root anchoring effect makes wheat more susceptible to strong wind and root lodging. For example, wheat in soils saturated with water suffered heavy lodging at 9.8–12.3 m/s wind speeds, as seen in Tancheng county, Shandong, in 2007 and in Zaozhuang city, Shandong and Bayinnaoer city of the Inner Mongolia Autonomous Region in 2013.

**Fig 5:**
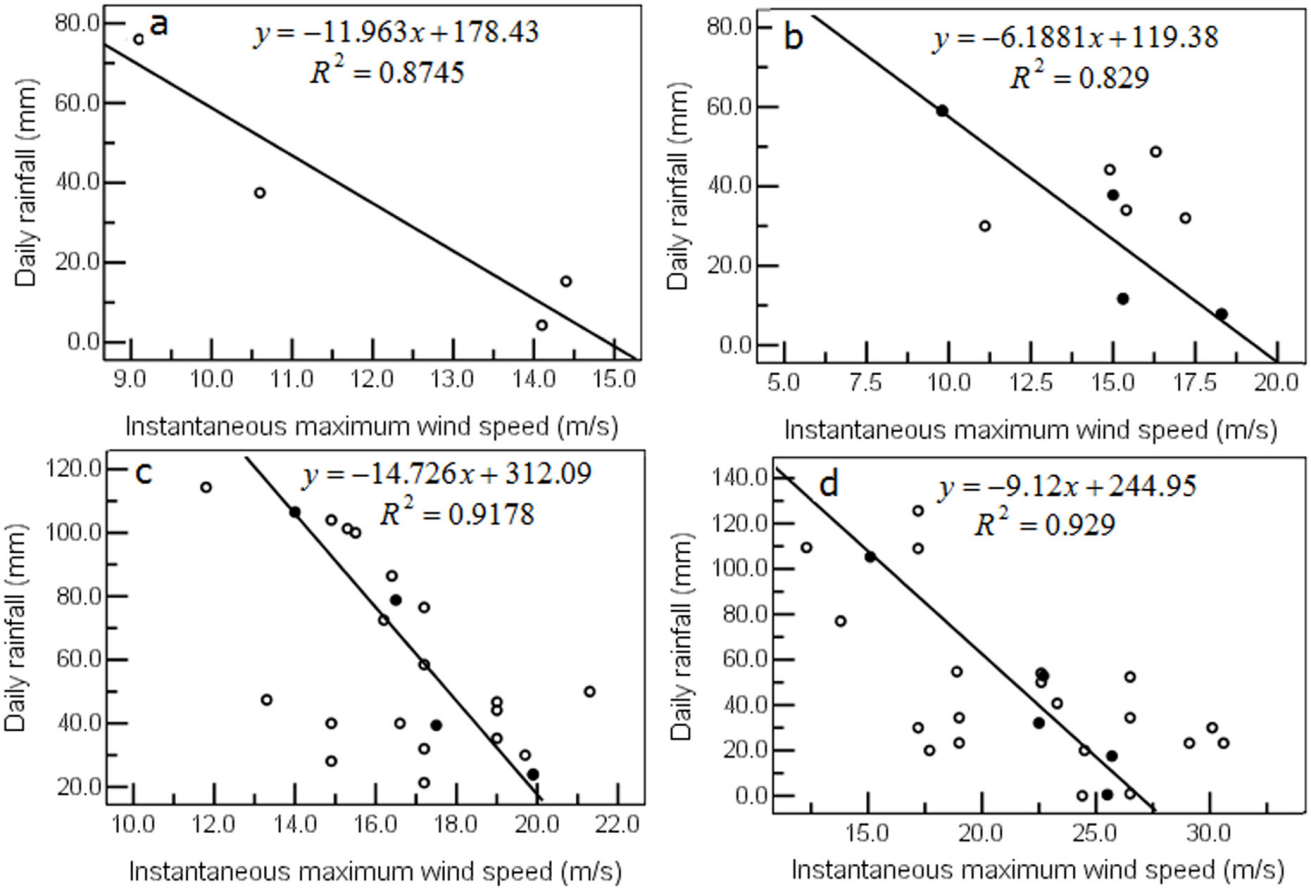
Interaction between daily rainfall and instantaneous maximum wind speed as it affected the level of severity of lodging. Sample values (○), Sample average values (●). a. Level 2. b. Level 3. c. Level 4. d. Level 5.

For quantitative research on the interaction between rainfall and instantaneous maximum wind speed as it affected lodging, the rainfall was categorized as follows: light (0–9.9 mm/day), moderate (10.0–24.9 mm/day), heavy (25.0–49.5 mm/day), torrential (50.0–99.0 mm/day), and heavy storm (above 100.0 mm/day). The average rainfall was then plotted against instantaneous maximum wind speed (Fig 5a–5d). Statistical results showed that there was a significantly negative relationship between the instantaneous maximum wind speed and average rainfall for the level 2–5 lodging, the correlation coefficient r was -0.935, -0.911, -0.958 and -0.964, respectively; the regression equation’s *R*^*2*^ was 0.8745, 0.829, 0.9178 and 0.929 (Fig 5a–5d), respectively. Using linear extrapolation and introducing different intensities of rainfall into the equations, the critical wind speed that would result in a given severity of lodging was calculated without rainfall or with varying intensities of rainfall. Without rainfall, instantaneous maximum wind speeds of 14.9 m/s, 19.3 m/s, 21.5 m/s, and 26.5 m/s, or maximum wind speeds of 9.4 m/s, 12.1 m/s, 13.5 m/s, and 16.7 m/s (equivalent to Force 5, 5–6, 6, and 7, respectively, on the Beaufort scale) resulted in severity levels of 2, 3, 4, and 5, respectively; *with* rainfall, similar levels of severity of lodging were reached at lower wind speeds: for example, the severity of lodging without rain at a given wind speed was the same as that at 95% of that wind speed when combined with light rain. The corresponding decrease in wind speed, which followed a linear pattern, that resulted in lodging of given severity, was as follows: 87% with moderate rain, 75% with heavy rain, and 49% with torrential rain.

#### Weather and lodging

Strong winds, continuous rain, and the two combined accounted for 8%, 19%, and 73% of the total instances of lodging examined in the present study, respectively. Apart from a few instances involving continuous rain, strong convectional weather was the most important type of weather to cause large-scale lodging: of the 52 instances examined, lodging caused by strong winds and by strong wind combined with heavy rain accounted for 42 times, or 81%, of the total.

The time at which the lodging occurred also followed a clear pattern. Of the 52 instances of lodging, 42 times, or 81%, occurred in the afternoon or the evening. The duration required for lodging depended on the weather. Lodging due to strong winds and that due to a combination of strong winds and heavy rainfall occurred quickly, within about 10 minutes to 2 hours. For example, heavy rains (52.4 mm) for half an hour with at least 10 minutes of Force 7–8 winds (instantaneous maximum wind speed of 28.3 m/s) resulted in lodging over more than 12 000 ha in Weishi county, Henan, on 17 May 2008, of which in nearly 6 667 ha the severity of lodging was Level 4 or higher; on 6 June 2009, heavy rain (50 mm), maximum wind speed of 16.2 m/s, and a hailstorm for 10 minutes led to lodging in nearly 540 ha in Luyi county, Henan. The duration of continuous rainfall that led to lodging, when not accompanied by strong winds, was usually longer, ranging from several hours to several days. These results indicate that strong convectional weather was the main type of weather for large-scale lodging in wheat.

### Type and distribution of lodging

**Fig 6:**
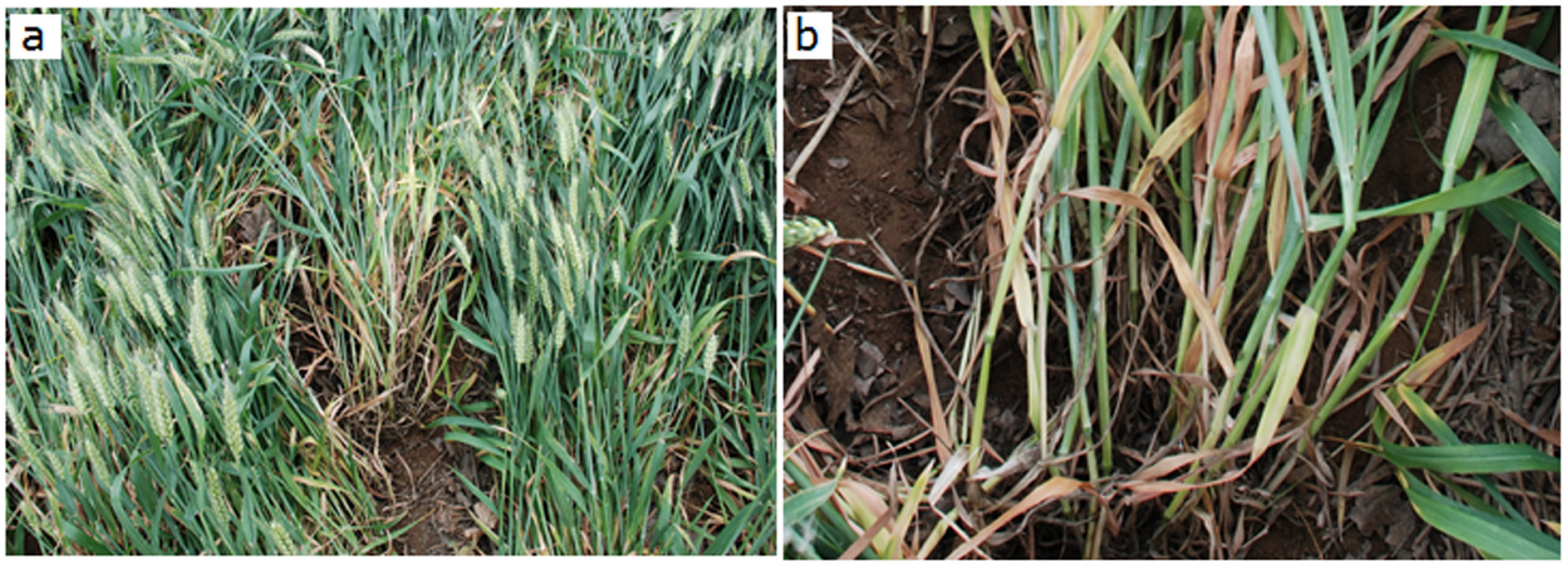
Lodging in Xinxiang city, Henan, in June 2014. a. A spot showing many affected plants. b. Close-up of a few plants.

To study a recent instance of lodging in wheat, the authors conducted a field investigation in the area around Xinxiang city, Henan, from May to June 2014. The area had suffered two instances of larger-scale lodging, on 1 May and 15 June. The field survey showed that the major type continued to be stem lodging (Fig 6a and 6b): strong winds coupled with heavy rainfall mostly result in stem lodging, whereas continuous rains alone mostly lead to root lodging. These findings helped in elucidating the phenomenon of lodging in greater detail.

At present, except for a few instances of wheat lodging, large-scale lodging in wheat occurs mainly in the Huanghuai basin of China’s main wheat-producing region. The distribution of the 52 instances of wheat lodging, by province, was as follows: Shandong, 22 instances; Hebei, 9; Henan, 5; Anhui, 4; Jiangsu and the Xinjiang Uyghur Autonomous Region, 3 each; Hubei and Shanxi, 2 each; and Heilongjiang and the Inner Mongolia Autonomous Region, 1 each.

The wind and rain, especially the wind speed is the most important external factors that cause the population lodging; the lodging results from the horizontal force exerted upon the population stalks by wind (wind load) more than the maximum bending moment that the population stalk is able to withstand at their base[1,7]. The research on large-scale wheat lodging critical wind speed, rainfall and their interaction could provide the basis for the establishment of wheat breeding objectives and breeding strategies.

Lodging is closely related to wheat plant own factors (for example stalk strength) and rainfall, wind speed and other external factors. Under the condition of the same wind and rain, the degree of the lodging is significantly negative related to the stalk strength. The stalk strength has close relationship with plant high, stem thickness, elastic, planting pattern, population density, but because a lot of research on these have been done [1–9], no longer discussed in this paper. Therefore, it is necessary to comprehensively consider both the wind, rain outside factors and internal factors such as plant stem strength on lodging effects in wheat breeding and production practice.

## Conclusions

Currently, large-scale lodging in China has three causes: strong winds, continuous rainfall, and strong winds combined with heavy rainfall; the last accounted for 73% of the 52 instances of large-scale lodging studied in the present paper. Strong convectional weather was the main type of weather that led to large-scale lodging. Wind speed was the dominant factor and rainfall was the auxiliary factor. The minimum wind speed that could cause large-scale lodging was closely related to rainfall. Without rainfall, the instantaneous minimum wind speed that resulted in lodging ranging in severity from slight to severe (Level 2 to Level 5) was 14.9 m/s, 19.3 m/s, 21.5 m/s, and 26.5 m/s, respectively (the corresponding maximum wind speeds being 9.4 m/s, 12.1 m/s, 13.5 m/s, and 16.7 m/s, equivalent to Force 5, 5–6, and 6–7 on the Beaufort scale); when accompanied by rainfall, the instantaneous minimum wind speed that resulted in lodging of the same severity decreased linearly with rainfall: with light rain, the wind speed was 95% of that which resulted in the same severity of lodging in the absence of rain, the corresponding decrease with moderate rain being 87%; with heavy rain, 75%; and with torrential rain, 49%. Shandong was the province that was most prone to lodging (in terms of both the extent and the severity of lodging), followed in that order by Hebei and Anhui. In breeding wheat for resistance to lodging, breeders should focus on strong winds together with heavy rainfall; such resistant varieties, to be worthwhile, should be able to withstand strong winds (equivalent to Force 6 or above on the Beaufort scale).

## Supporting Information

S1 Table No. of instances, Instantaneous maximum wind speed (m/s), Daily rainfall (mm) and Severity of lodging. Meteorological data in the table were obtained from two sources: (1) the China Meteorological Data Sharing Network http://cdc.cma.gov.cn/; (2) Huayun Information Technology Engineering Limited company (paid service).
(DOC)
