# Supplementary material for: Influence of Speed and Rainfall on Large-Scale Wheat Lodging from 2007 to 2014 in China
Source: PLoS One. 2016 Jul 1;11(7):e0157677. doi: 10.1371/journal.pone.0157677 (PMC4930167; doi:10.1371/journal.pone.0157677)
Supplement: S1 Table — Meteorological data in the table were obtained from two sources: (1) the China Meteorological Data Sharing Network http://cdc.cma.gov.cn/; (2) Huayun Information Technology Engineering Limited company (paid service). (DOC) [file pone.0157677.s001.doc]

**Supporting Information**

**S1 Table** No. of instances, Instantaneous maximum wind speed (m/s), Daily rainfall (mm) and Severity of lodging

| **No. of instances** | **Instantaneous maximum wind speed (m/s)** | **Daily rainfall (mm)** | **Severity of lodging** |
| --- | --- | --- | --- |
| **1** | 13.3 | 47.4 | 4 |
| **2** | 24.4 | 0.0 | 5 |
| **3** | 17.2 | 58.5 | 4 |
| **4** | 26.5 | 52.4 | 5 |
| **5** | 17.2 | 21.3 | 4 |
| **6** | 22.6 | 50.0 | 5 |
| **7** | 29.1 | 23.2 | 5 |
| **8** | 16.2 | 72.5 | 4 |
| **9** | 30.1 | 30.0 | 5 |
| **10** | 17.7 | 20.0 | 5 |
| **11** | 26.5 | 34.4 | 5 |
| **12** | 19.9 | 23.9 | 4 |
| **13** | 30.6 | 23.2 | 5 |
| **14** | 15.4 | 34.0 | 3 |
| **15** | 26.5 | 0.9 | 5 |
| **16** | 22.6 | 54.0 | 5 |
| **17** | 9.1 | 80.0 | 2 |
| **18** | 14.9 | 28.1 | 4 |
| **19** | 17.2 | 125.6 | 5 |
| **20** | 17.2 | 30.0 | 5 |
| **21** | 14.1 | 4.3 | 2 |
| **22** | 19.0 | 34.4 | 5 |
| **23** | 14.4 | 15.3 | 2 |
| **24** | 16.3 | 48.7 | 3 |
| **25** | 10.6 | 37.5 | 2 |
| **26** | 24.5 | 20.0 | 5 |
| **27** | 11.8 | 114.3 | 4 |
| **28** | 11.1 | 30.0 | 3 |
| **29** | 19.0 | 46.7 | 4 |
| **30** | 17.2 | 109.0 | 5 |
| **31** | 18.9 | 54.7 | 5 |
| **32** | 14.9 | 44.2 | 3 |
| **33** | 15.3 | 101.3 | 4 |
| **34** | 10.2 | 133.0 | 5 |
| **35** | 23.3 | 40.7 | 5 |
| **36** | 19.0 | 44.1 | 4 |
| **37** | 14.9 | 104.0 | 4 |
| **38** | 19.7 | 30.0 | 4 |
| **39** | 16.6 | 40.0 | 4 |
| **40** | 15.5 | 100.0 | 4 |
| **41** | 19.0 | 23.3 | 5 |
| **42** | 14.9 | 40.0 | 4 |
| **43** | 17.2 | 32.0 | 3 |
| **44** | 17.2 | 32.0 | 4 |
| **45** | 17.2 | 76.5 | 4 |
| **46** | 19.0 | 35.3 | 4 |
| **47** | 13.8 | 77.0 | 5 |
| **48** | 9.8 | 59.0 | 3 |
| **49** | 18.3 | 7.8 | 3 |
| **50** | 21.3 | 50.0 | 4 |
| **51** | 16.4 | 86.4 | 4 |
| **52** | 15.3 | 11.7 | 3 |

Meteorological data in the table were obtained from two sources:

(1) The China Meteorological Data Sharing Network http://cdc.cma.gov.cn;

(2) Huayun Information Technology Engineering Limited company (paid service).
